# Imaging predictors of hemorrhagic progression of a contusion after traumatic brain injury: a systematic review and meta-analysis

**DOI:** 10.1038/s41598-024-56232-w

**Published:** 2024-03-12

**Authors:** Jie Peng, Tao Luo, Xiaoyu Li, Bin Li, Yuan Cheng, Qin Huang, Jun Su

**Affiliations:** 1 Department of Neurosurgery, The People’s Hospital of Nanchuan, Chongqing, 408400 China; 2 Department of Neurosurgery, The Second Affiliated Hospital of Chongqing Medical University, Chongqing, 400010 China

**Keywords:** Traumatic brain injury, Hemorrhagic progression of a contusion, Imaging features, Meta-analysis, Imaging predictors, Neuroscience, Neurology

## Abstract

The hemorrhagic progression of a contusion (HPC) after Traumatic brain injury (TBI) is one of the important causes of death in trauma patients. The purpose of this meta-analysis was to evaluate the predictive effect of imaging features of Computed tomography (CT) on HPC after TBI. A comprehensive systematic search was performed using PubMed, EMBASE, and WEB OF SCIENCE databases to identify all relevant literature. A total of 8 studies involving 2543 patients were included in this meta-analysis. Meta-analysis showed that subarachnoid hemorrhage (OR 3.28; 95% CI 2.57–4.20), subdural hemorrhage (OR 4.35; 95% CI 3.29–5.75), epidural hemorrhage (OR 1.47;95% CI 1.15–1.89), contrast extravasation (OR 11.81; 95% CI 4.86–28.71) had a predictive effect on the occurrence of HPC. Skull fracture (OR 1.64; 95% CI 0.84–3.19) showed no statistical significance, and midline displacement > 5 mm (OR 4.66; 95% CI 1.87–11.62) showed high heterogeneity. The results of this meta-analysis showed that some imaging features were effective predictors of HPC after TBI. Well-designed prospective studies are needed to more accurately assess the effective predictors of HPC after TBI.

## Introduction

Traumatic brain injury (TBI) is the important cause of death for trauma victims, mostly due to traffic accidents, followed by falls from a height. About 2.8 million cases of TBI occurred in the United States in 2013, while the incidence of TBI in Europe is 235 per 100,000^1–3^. Some studies have found that 35–65% of patients with TBI have hemorrhagic progression of contusion (HPC), which increases the risk of aggravation of clinical symptoms by 5 times. Therefore, this is an important cause of disability and death of trauma victims^4–8^. TBI has caused an increasing socio-economic burden in the world. The main reason is that HPC after TBI can not be predicted in time, and the patients cannot are managed hierarchically. If effective hierarchical management can be achieved, intervention measures can be put in place earlier to improve prognosis and allocate medical resource more rationally^9,10^.

In the past few decades, many studies have explored the predictive value of hematological and biochemical indicators for HPC, and relatively few studies have analyzed the predictive value of imaging features. Studies have found that platelet count, coagulation function test and plasma D-dimer have a predictive effect on HPC^11–16^. Hematological and biochemical parameters and imaging examinations are of great value in the diagnosis and prognosis of HPC. Computed tomography (CT) examination is the preferred imaging examination for the diagnosis of TBI, which can identify a variety of hematomas more quickly. Therefore, this study mainly discusses the imaging features of CT. Some studies have found that some imaging features is an independent predictor of HPC, such as subarachnoid hemorrhage (SAH), subdural hemorrhage (SDH), epidural hemorrhage (EDH) and contrast extravasation (CE)^17–24^. Therefore, the purpose of this study is to further systematically review and clarify the predictive value of some imaging features for HPC, and to provide a clinical basis for prediction of HPC after TBI.

## Method

Systematic reviews and meta-analyses were performed according to the guidelines for systematic reviews and meta-analyses (PRISMA)^25^. This study was prospectively registered in the PROSPERO system evaluation database (CRD42022350212).

### Literature search

A comprehensive literature search was performed in PubMed, EMBASE, and Web of Science databases to assess the association between imaging features and HPC after TBI. The search terms included ' cerebral contusion ', 'brain injury ', ' traumatic brain injury ', ' hemorrhagic progression of contusion ', ' extravasation of contrast agent ', ' leakage sign ', ' spot sign ' and their synonyms. The search time is from January 2007 to April 2022.

## Inclusion and exclusion criteria

We selected articles according to the following inclusion criteria: (1) Head imaging examination with clear diagnosis of TBI and reexamination; (2) Outcome index was HPC; (3) The data of the selected articles can be used to calculate the OR value and 95% confidence interval; (4) The included population included mild, moderate and severe TBI patients (excluding only one or two groups). The exclusion criteria are as follows: (1) The same author is excluded; (2) Imaging factors are not the main object of study; (3) Reviews, conference reports and case reports are excluded. We reviewed the selected articles and summarized the relationship between imaging features and HPC in 34 studies, of which 8 studies met the inclusion criteria.

### Data abstraction

The list of articles generated by the literature search was manually reviewed by two investigators (Peng and Luo). The following information is included: author, age, country, sample size, gender and hemorrhagic progression volume. Any differences will be resolved through consultation between the two authors.

### Assessment of risk of bias and quality

Two authors (Peng and Luo) independently assessed the quality of the included article by the Newcastle–Ottawa Quality Assessment Scale (four for quality of selection, two for comparability, and three for quality of outcome and adequacy of follow-up). The quality of the study was classified as low (below 6), moderate (7), and high (8–9). Conflicts were resolved by consensus between the two authors.

### Statistical analysis

All statistical analyses were performed using Review Manager Version 5.4 software. For dichotomous variables, we calculated the odds ratio (OR) with a 95% confidence interval (CI). Whenever I^2^ was less than 50%, the fixed-effects model results were used; otherwise, the random-effects model was preferred. For those with high heterogeneity, sensitivity analysis and subgroup analysis can be performed to evaluate the stability of the results.

## Results

**Figure 1:**
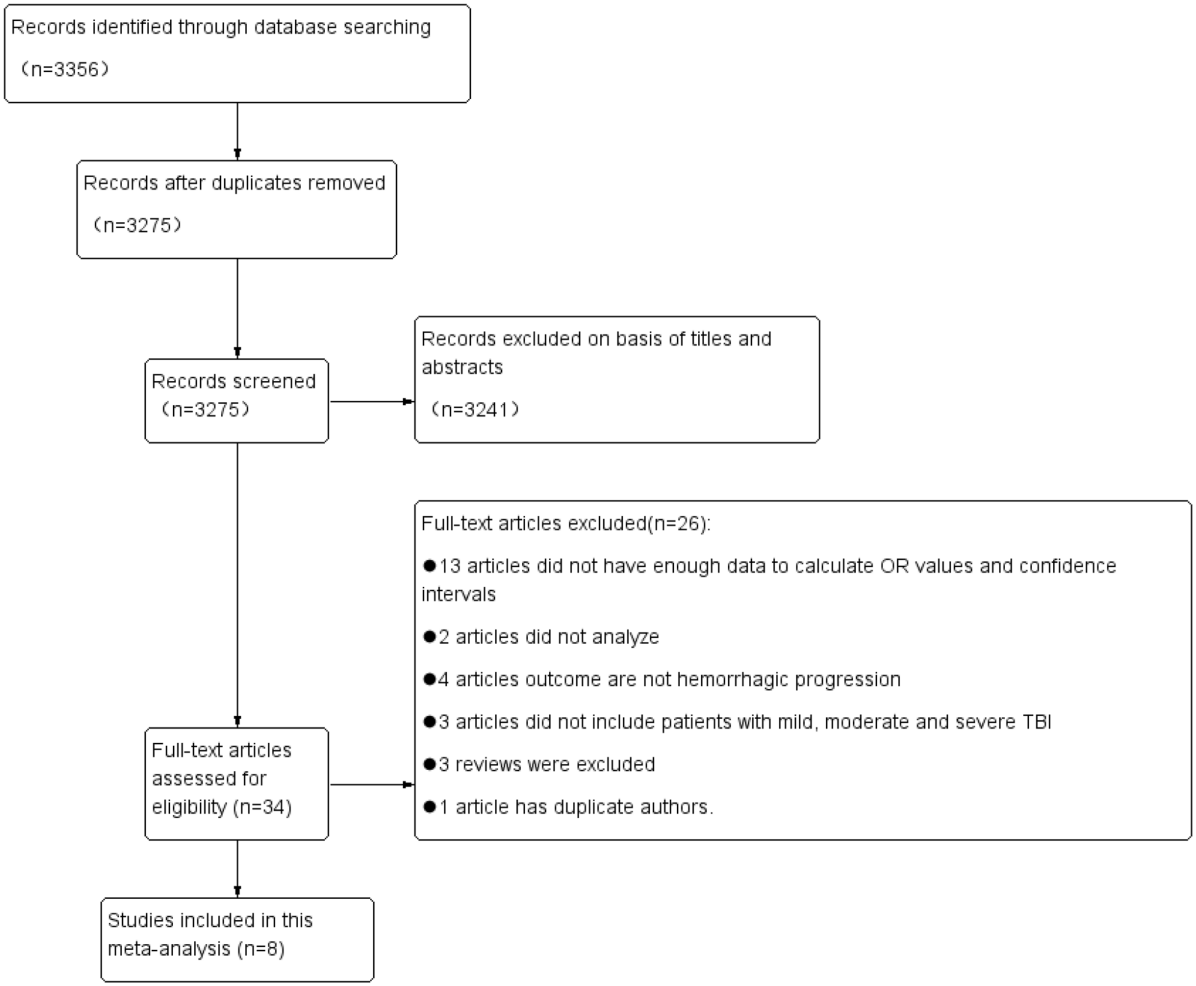
Flowchart of the article search performed.

The process and results of retrieving the article are shown in Fig. 1. A total of 3356 articles were retrieved using PubMed, EMBASE, and Web of Science databases. After deleting duplicate articles, 3275 articles remained. After reading the title and abstract, a total of 3241 articles were excluded because they did not meet the topic. After reading the full text of the remaining 34 articles, 8 articles were retained (Fig. 1). Among them, 26 articles were excluded for the following reasons: 13 articles did not have enough data to calculate the OR value and 95% confidence interval; 2 articles did not analyze the imaging features; the outcome index of 4 articles was not HPC; 3 articles did not include patients with mild, moderate and severe TBI; 3 reviews were excluded; 1 article was repeated by the author.

### Study characteristics

The characteristics of the included studies are shown in Table 1. All studies were published between 2007 and 2022. The diagnosis of TBI patients is confirmed by a clear history of TBI and imaging examination. Author, year, location, sample size, average age, gender, and hemorrhagic progression volume are shown in Table 1. Four studies were case–control studies and four studies were cohort studies.

**Table 1:** Summary of characteristics of the included studies.

References	Location	Sample size	Mean age	Gender distribution	hemorrhagic progression volume
Tong et al.^18^	China	630	44	M 458;W 172	> 25%
Huang et al.^21^	China	22	59.4	M 14;W 18	> 5 ml or > 30%
Yuan et al.^20^	China	468	HPC:51.87;NO HPC:42.84	M 364;W 104	> 25%
Letourneau-Guillon et al.^22^	Canada	60	CE:50;NO CE:56	M 46;W 14	> 12 ml and > 33%
Wang et al.^19^	China	132	HPC:51.43;NO HPC: 46.77	M 94;W 38	> 25%
Rosa et al.^23^	Brazil	121	Not clear	M 83;W 13	> 6 ml or > 33%
Rehman et al.^11^	Pakistan	246	M:40.38;W:34.29	M 212;W 34	> 30%
Sheng et al.^17^	China	889	HPC:47.69;NO HPC: 53.12	M 683;W 206	> 5 ml and > 33%

*HPC* hemorrhagic progression of a contusion; *CE* contrast extravasation; *M* man; *W* woman.

### Risk of bias assessment

The Newcastle–Ottawa quality assessment score included in the study was between 8 and 9 points. All the 8 studies were of high quality. Four case–control studies scored 8 (Table 2). In the cohort study, 2 articles scored 9 points and 2 articles scored 8 points (Table 3).

**Table 2:** Results of quality assessment using the NOS for the case–control study.

References	Selection	Comparability	Outcome	Total
Tong et al.^18^	***	**	***	8
Huang et al.^21^	***	**	***	8
Wang et al.^19^	***	**	***	8
Sheng et al.^17^	***	**	***	8

*NOS* : the Newcastle–Ottawa scale.

**Table 3:** Results of quality assessment using the NOS for the cohort studies.

References	Selection	Comparability	Outcome	Quality score
Huang et al.^21^	****	*	***	8
Letourneau-Guillon et al.^22^	***	**	***	8
Rosa et al.^23^	****	**	***	9
Rehman et al.^11^	****	**	***	9

*NOS* : the Newcastle–Ottawa scale

### Data analysis

**Figure 2:**
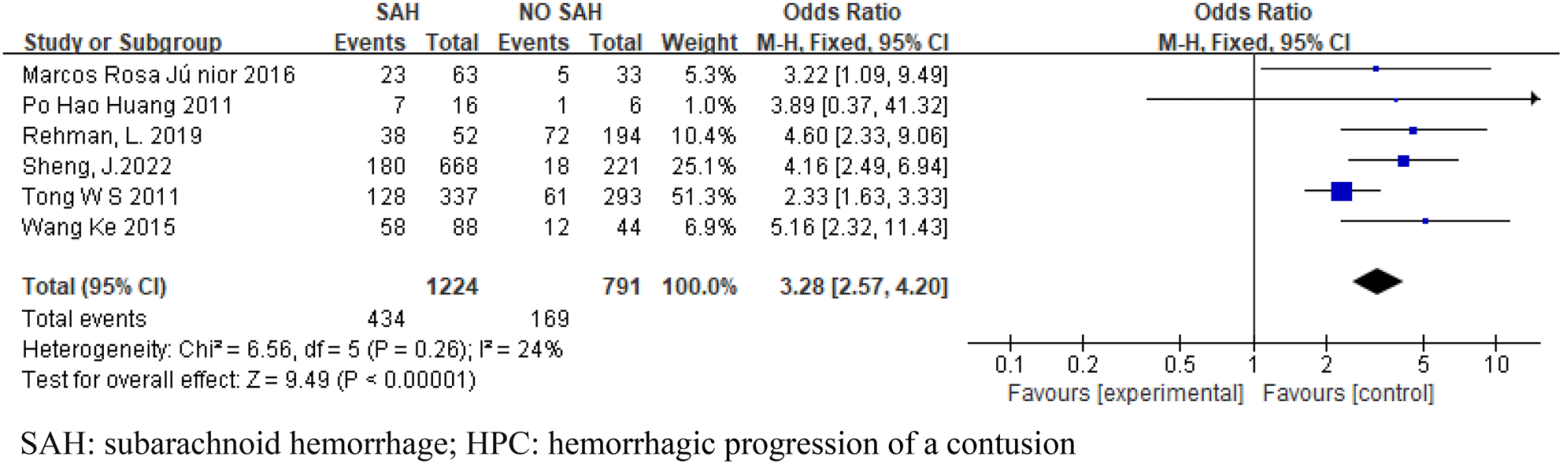
Forest plot analyzing the predictive effect of SAH for HPC. SAH: subarachnoid hemorrhage; HPC: hemorrhagic progression of a contusion.

**Figure 3:**
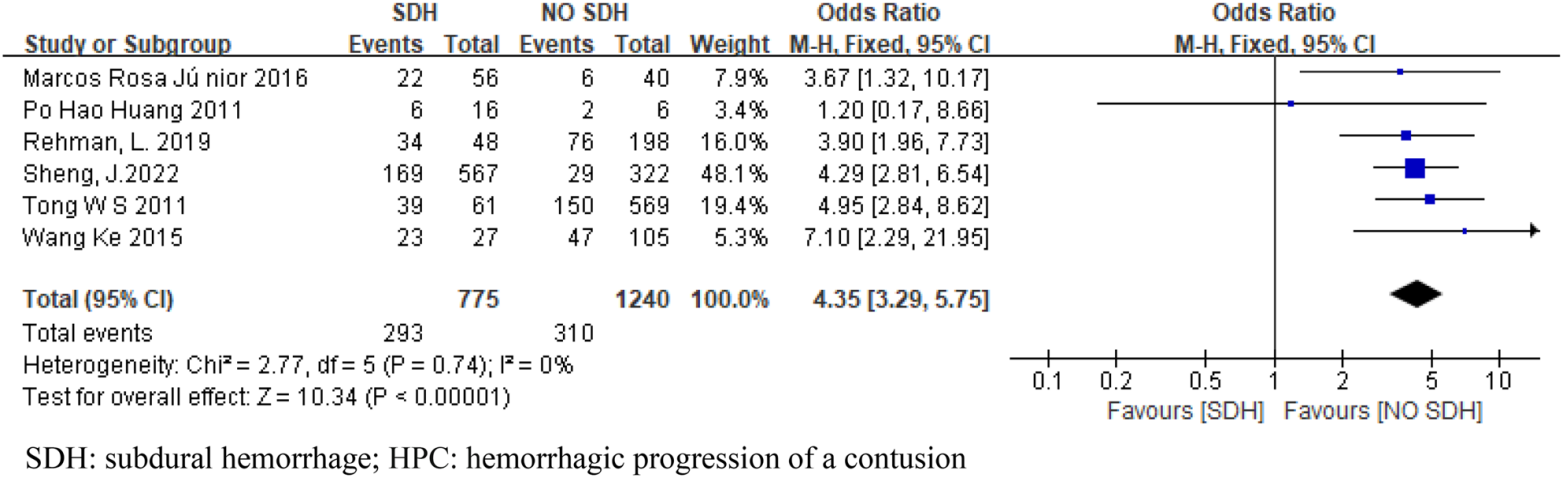
Forest plot analyzing the predictive effect of SDH for HPC. SDH: subdural hemorrhage; HPC: hemorrhagic progression of a contusion.

**Figure 4:**
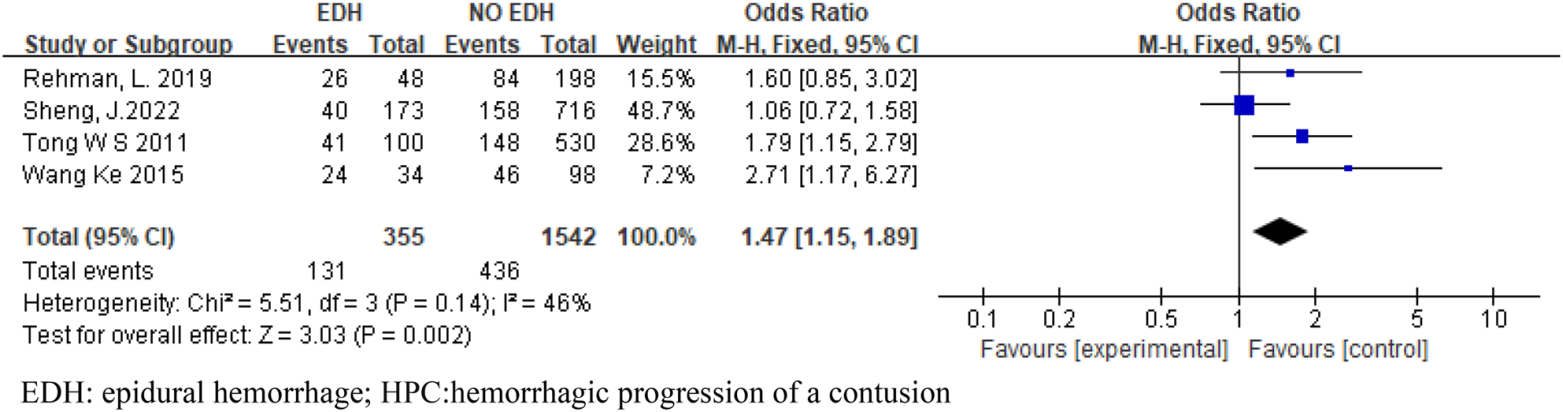
Forest plot analyzing the predictive effect of EDH for HPC. EDH: epidural hemorrhage; HPC:hemorrhagic progression of a contusion.

**Figure 5:**
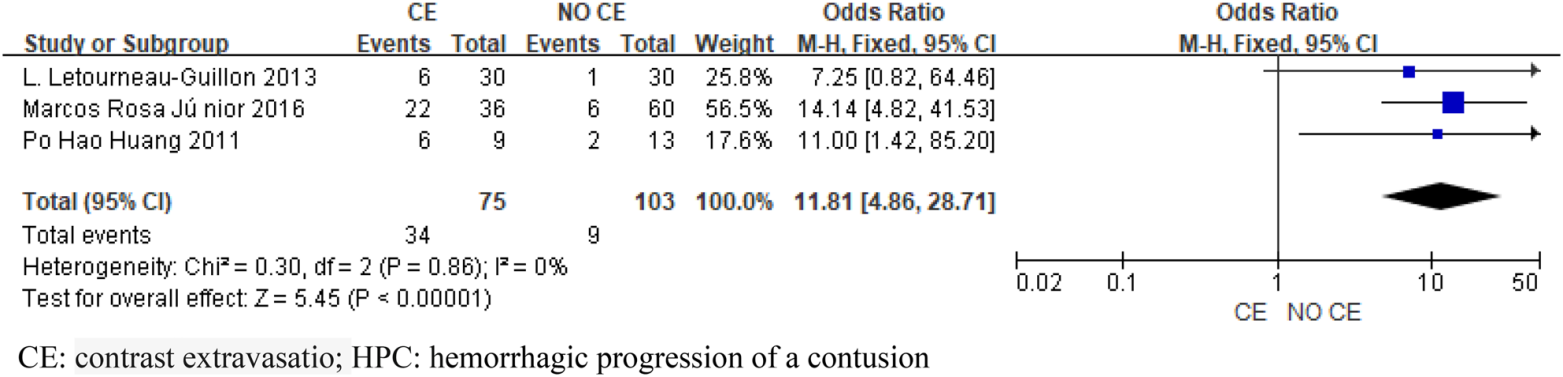
Forest plot analyzing the predictive effect of CE for HPC. CE: contrast extravasatio; HPC: hemorrhagic progression of a contusion.

Eight articles were included in this meta-analysis, and 2543 patients were included. Six articles included SAH and SDH. Four articles were included in EDH. Four articles included skull fractures. Three articles were included in CE. Two articles included midline shift. The pooled results showed that SAH (OR 3.28; 95% CI 2.57–4.20; I^2^ = 24%; Fig. 2), SDH (OR 4.35; 95% CI 3.29–5.75; I^2^ = 0%; Fig. 3), EDH (OR 1.47 ; 95% CI 1.15–1.89 ; I^2^ = 46% ; Fig. 4), CE (OR 11.81; 95% CI 4.86–28.71; I^2^ = 0%; Fig. 5) all have a certain predictive effect on HPC. The above results were statistically significant (*P* < 0.05). The heterogeneity results were not high.

**Figure 6:**
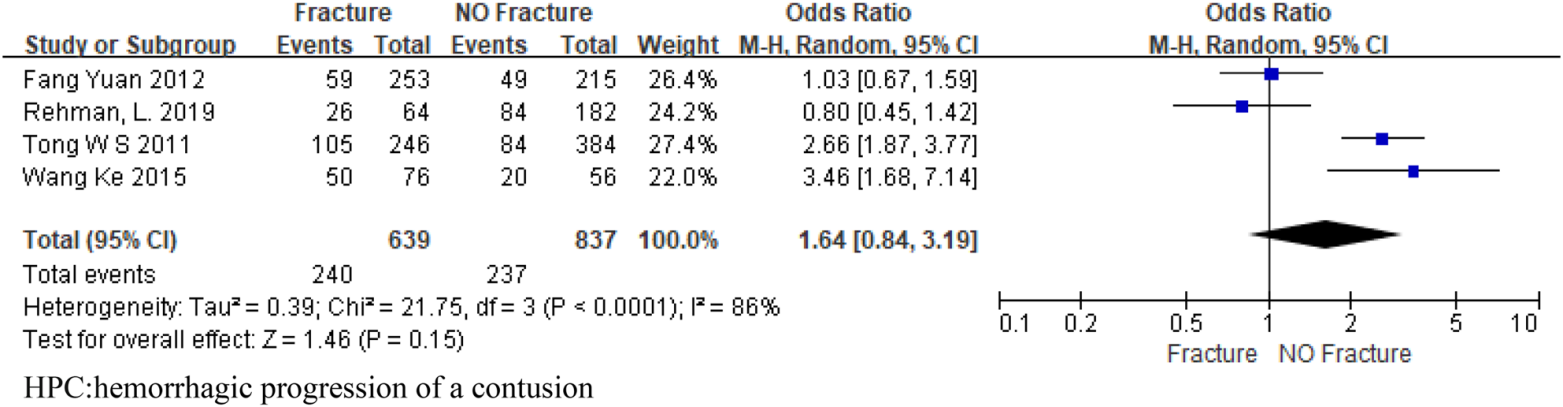
Forest plot analyzing the predictive effect of skull fracture for HPC. HPC:hemorrhagic progression of a contusion.

**Figure 7:**
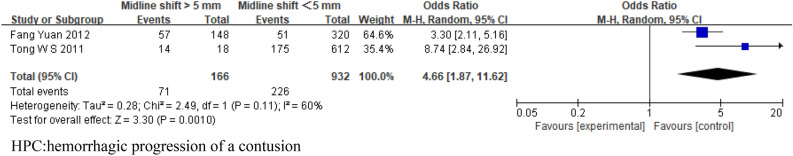
Forest plot analyzing the predictive effect of midline shift > 5 mm for HPC. HPC:hemorrhagic progression of a contusion.

Four articles analyzed the predictive effect of skull fracture on HPC, and a total of 1476 patients were included. The pooled OR was 1.64 ([95% CI 0.84, 3.19]. I^2^ = 86.0%; Fig. 6), the results showed no statistical significance. Two studies have shown the predictive effect of midline shift > 5 mm on HPC. The results showed that midline shift > 5 mm was statistically significant (OR 4.66; 95% CI 1.87–11.62; I^2^ = 60%; Fig. 7). The reason for the high heterogeneity may be the insufficient number of included studies and the large difference between the two sample sizes.

## Discussion

To our knowledge, there is no meta-analysis to systematically summarize and explore the predictive effect of imaging features on HPC after TBI. In TBI patients, HPC mostly occurs within 24 h before onset, and rarely occurs 3–4 days after onset^4,26,27^. Due to the high incidence of HPC after TBI, imaging features predict that HPC is crucial for the treatment of TBI patients. The purpose of this meta-analysis is to further clarify the predictive value of imaging features of CT. The results of this meta-analysis show that some imaging features are effective predictors of HPC. Among them, CE, SAH, SDH, and midline shift have obvious predictive effects. EDH has less predictive effect than the above imaging features. There is no statistical significance in skull fracture, and the heterogeneity of midline shift in forest map is high.

There is a lack of standardized definition of HPC in previous studies. When the amount of bleeding exceeds a threshold or the increase in the range of contusion is defined as HPC, but this threshold and the range of contusion increase are not uniform. This is the main reason for the difference in the incidence of contusion hemorrhage. Part of the reason is caused by the calculation method of assessing the volume of contusion. The previous traditional mechanism of HPC is coagulation dysfunction after TBI, but the occurrence of HPC in some studies does not seem to be related to coagulation dysfunction. In recent years, it has been found that ' traumatic penumbra ' may better explain the mechanism of HPC after TBI, but there are few studies on imaging features^17–23^.

Many studies have examined the predictive effect of hematological examinations such as coagulation function and platelets .TBI can cause small vessel injury and coagulation mechanism disorder, leading to HPC. Platelets can promote hemostasis, accelerate coagulation, and participate in the repair of vascular endothelium. Plasma D-dimer is an important indicator of coagulation function, reflecting disseminated intravascular coagulation.These indicators have a certain predictive effect on HPC^11–16^.

SAH causes extravasation of erythrocyte decomposition products and cerebral vasospasm, leading to ischemia and necrosis of the vascular wall. Extravasated red blood cells may trigger an inflammatory cascade^5,19,28^. SDH can reflect bridging vein rupture and venous sinus injury, with persistent bleeding, aggravating edema and compression effect^5,17^. After skull fracture, meningeal artery bleeding and veins can be damaged, resulting in persistent bleeding. Skull fracture may have middle meningeal artery injury, plate barrier bleeding and venous sinus rupture. The continuous bleeding of these arteries and veins leads to HPC^19^. The mechanism of HPC after EDH may be related to arteriovenous hemorrhage, secondary damage to the underlying cortex, and hematoma compression effect. Midline shift > 5 mm may also be an effective predictor of HPC. Midline shift > 5 mm shows a wide range of contusion, laceration, endothelial cell injury, swelling, vasospasm and microvascular injury^4,14,28^. CE shows that cerebrovascular endothelial cell damage after TBI patients, leading to active bleeding^21,24^. CE has been widely studied in spontaneous intracerebral hemorrhage (ICH) and is considered to be a predictor of cerebral hemorrhage expansion, adverse outcomes and mortality. Recent studies have shown that CE has predictive value for HPC after TBI, so this study included CE^29–31^.

In this penumbra, metabolic function is abnormal, and brain tissue is more vulnerable to secondary damage. Traumatic penumbra after TBI activates specific protein 1 and nuclear factor. Subsequently, the expression of sulfonylurea receptor 1 changed the expression of aquaporin 4, increased the permeability of the blood–brain barrier, formed vascular edema, endothelial cell death and capillary rupture, resulting in contrast agent exudation. Some studies have found that the loss of tight junction (TJ) protein and the increase of endocytosis of endothelial cells (EC) may also be related to the change of blood–brain barrier (BBB) permeability^32–35^. Dot signs and CE were more common in patients with spontaneous intracerebral hemorrhage, but CE was more common in TBI patients. Studies have shown that dot signs are vascular injury or perforation after spontaneous intracerebral hemorrhage. However, vascular injury or perforation directly caused by TBI is less likely. Secondary BBB rupture occurs within a few days after injury. In patients with TBI, secondary BBB damage, ischemic necrosis or perforation caused by ischemia or reperfusion injury takes time, which may explain the reason why there is no spot sign in the early stage of TBI^21,24,36–42^.

The traditional mechanism is that coagulation disorders lead to continuous or delayed bleeding of ruptured microvessels during primary injury, so there have been many studies on hematological and biochemical indicators^5,36^. However, the theory of ' traumatic penumbra suggests that vascular injury may be the main cause of HPC, and imaging features can show persistent bleeding after vascular injury, so some imaging features may be effective predictors. In recent years, studies proposed the multihematoma fuzzy sign after TBI. Multihematoma fuzzy sign usually refers to the contusion area with blood clots and fresh liquid blood. The presence of fresh liquid blood indicates that there may be ongoing active bleeding^17^.

This meta-analysis showed that skull fracture was not statistically significant, and the midline shift was > 5 mm shows high heterogeneity. There is no statistical significance of skull fracture may be due to the following reasons: (1) Rehman et al.^11^ only included patients with blunt brain injury, excluding penetrating brain injury. Because penetrating brain injury is more likely to have HPC than blunt brain injury, and this study has a small sample size and is likely to cause bias. (2) Skull fractures include linear fractures, depressed fractures, comminuted fractures and cavernous fractures. Yuan et al.^20^ only included linear fractures, but linear fractures are less likely to deteriorate than other fractures, and the risk of HPC is relatively small. Sensitivity analysis showed that the reason for the high heterogeneity of the midline shift > 5 mm may be related to the small number of included articles and the large difference in the sample size of the included studies^18,20^.

Although this meta-analysis shows that some imaging features are predictors of HPC, there are still some limitations: (1) Due to the small number of articles included, publication bias cannot be analyzed. Half of the included studies were case–control studies, and the quality of the literature was insufficient, which affected the accuracy of the results. (2) Hematoma volume after TBI, multiple hematoma blur sign, TBI impact location, the first CT examination time may be effective predictors^11,17,19^. Due to the lack of the number of studies, it is impossible to explore the relationship with HPC. (3) HPC has no clear definition, and the cut-off values in definition are different. Oertel described the definition of HPC, including EDH, SDH, SAH and intracerebral hematoma^37^. Alahmadi et al. proposed a rough definition of HPC and described the prognosis and risk factors of HPC^43–48^. Sheng J established a predictive model for acute traumatic intracerebral hematoma expansion^17^. (4) This study included people with mild, moderate and severe TBI, but severe TBI may be more prone to hemorrhagic injury. Because the included articles did not divide the population into mild, moderate and severe groups, we could not conduct a comparative study separately, which also led to some bias in the results of this study.

For the management of TBI patients, timely prediction of HPC is very important. Imaging examination and hematological and biochemical parameters have predictive value for the occurrence of HPC, and some imaging features can significantly predict the occurrence of HPC. The establishment of a risk assessment system based on imaging features may be more accurate in predicting the occurrence of HPC in TBI patients. The study of Randall Z. Allison et al. established a scoring system to predict the occurrence of HPC through imaging features. Studies have analyzed the relationship between imaging features and HPC in TBI patients, but there is no meta-analysis system to study the relationship. This study further demonstrated the predictive value of imaging features in predicting HPC in TBI, and provided further evidence for the establishment of imaging scoring system in the future.

## Conclusion

The results of this meta-analysis show that some imaging features are effective predictors of HPC, and some mechanisms can explain these results, but more research is needed to explain the mechanism of HPC. High-quality cohort studies are needed to further demonstrate the predictive effect of imaging features on HPC.

## Data Availability

All data generated or analyzed during this study are included in these published articles.
